# Analysing the administration of an intermediate level of outpatient palliative care in Germany and developing recommendations for improvement (Polite): A study protocol for a mixed-methods study

**DOI:** 10.1371/journal.pone.0256467

**Published:** 2021-09-02

**Authors:** Stephanie Stiel, Katharina van Baal, Rojda Ülgüt, Jona T. Stahmeyer, Nils Schneider

**Affiliations:** 1 Institute for General Practice and Palliative Care, Hannover Medical School, Hanover, Germany; 2 Health Services Research Unit, AOK Niedersachsen, Hannover, Germany; PLoS ONE, UNITED STATES

## Abstract

**Background:**

To date, there has been no systematic research on the intermediate level service (level 2) in outpatient palliative care that was introduced in Germany in 2017. Accordingly, the Polite research project aims at: (1) investigating the current state of level 2 palliative care and (2) developing recommendations for its optimisation.

**Methods:**

The multi-perspective, observational study will follow a mixed-methods approach across two study phases. In phase 1a, quantitative routinely collected data from a statutory general local health insurance provider will be used to identify patients who received level 2 or other outpatient palliative care in the years 2017–2019. In phase 1b, a questionnaire will be sent to all registered general practitioners (GPs) in Lower Saxony to collect information on the number and characteristics of physicians offering level 2 palliative care. In phase 1c, a quantitative, standardised online questionnaire for teams providing specialised outpatient palliative care will be administered to assess the interfaces of level 2 palliative care. In phase 2a, the results from phases 1a–c will be discussed in an expert workshop with the aim of developing ideas to adapt and optimise level 2 outpatient palliative care. Finally, in phase 2b, the empirically derived recommendations from phases 1 and 2a will be agreed upon via a multi-round Delphi survey involving experts with sufficient influence to promote the project results and recommendations nationally.

**Discussion:**

The results of the project will facilitate the optimisation of outpatient palliative care, as well as its administration, nationwide.

**Trial registration:**

The study was registered in the German Clinical Trials Register (Deutsches Register Klinischer Studien) (Registration N° DRKS00024785); date of registration: 06^th^ May 2021) and is searchable under the International Clinical Trials Registry Platform Search Portal of the World Health Organization, under the German Clinical Trials Register number.

## Introduction

### Background

According to sociodemographic trends, it is estimated that at least 23.3% of German residents will be over 67 years old in 2030 [1]. The absolute number of German residents over the age of 80 years is expected to increase from 4.4 million in 2013 to more than 9 million in 2060 [1]; this projected figure corresponds to approximately 12–13% of future population estimates. Rising morbidity and health care costs among German’s elderly population are also expected [2].

Current estimates of need suggest that 40.7–96.1% of dying people could benefit from palliative care. An analysis of all deaths in 2013 in Germany found an estimated need of 78% across all age groups [2]. In general, approximately 80–90% of people requiring palliative care can be adequately cared for through generalist outpatient palliative care (level 1) [2]. The European Association for Palliative Care (EAPC) white paper on standards and norms for hospice and palliative care in Europe states: ‘Whereas a palliative care approach will provide adequate care for many of these patients, it has been estimated that at least 20% of cancer patients and 5% of non-cancer patients require specialist palliative care in the last year of their life’ [3]. Although palliative care offerings in Germany have recently expanded and the number of service providers has increased, there is still a great need for palliative care, especially in rural areas [4].

In Germany, outpatient and inpatient palliative care has undergone very dynamic development to respond to sociodemographic changes and palliative care needs. Important milestones in the political effort to improve palliative care in Germany have included the introduction of specialist outpatient palliative care (level 3) in the Social Code Book V (SGB V) in 2007 [5]; the inclusion of palliative care within the statutory health insurance system of general patient care in the Hospice and Palliative Care Act of December 2015 [6]; and the expansion of the Uniform Value Scale (‘Einheitlicher Bewertungsmaßstab’, EBM) to include generalist outpatient palliative care (level 1) by general practitioners (GPs) in 2013 [4, 7]. In 2016, during the implementation of the Hospice and Palliative Care Act, an agreement was reached for the establishment of an intermediate level of generalist outpatient palliative care (level 2) [8, 9].

The new legislation aimed at introducing level 2 generalist outpatient palliative care as an intermediate step between basic level 1 care and level 3 specialist outpatient palliative care in the pyramid-like care structure [5, 8]. In a white paper on standards and norms, for example, the EAPC described and recommended the graduation of: 1) a palliative care approach, 2) general palliative care and 3) specialist palliative care [3]. This three-level system of outpatient palliative care was already a feature of several international health care systems.

The proposed expansion was based on the assumption that some seriously ill and dying people were under-served by level 1 palliative care, but not good candidates for level 3, due to a lack of complex symptoms, among other reasons. Thus, level 2 palliative care was proposed to treat patients with an incurable, progressive and advanced disease for which curative therapy was no longer possible or desired, but who still required interdisciplinary and comprehensive palliative medical treatment, appropriate for their stage of disease [10, 11]. Level 2 care could be administered in outpatient settings, such as patients’ private homes, as well as assisted living facilities, inpatient nursing homes, day hospices and inpatient hospices. However, while level 1 palliative care can be provided and billed by all GPs, only GPs with special qualification could offer palliative care at levels 2 or 3.

The overarching goals of level 2 outpatient palliative care were: a) to improve the care of seriously ill patients (in all age groups) with palliative care needs/wishes in their last phase of life, b) to improve the provision of outpatient palliative care, c) to allow patients to die in the environment of their choice and d) to establish needs-based structures, coordination and on-call care [10, 11]. These goals were considered sufficient to address the essential problem areas of end-of-life care for people in Germany.

### Study aims

To date, there have been no systematic evaluations of level 2 palliative care since it was first implemented in Germany in 2017. Scientific research is needed to: 1) uncover the barriers to and facilitators of level 2 palliative care, 2) provide empirical data on the administration of this new form of outpatient palliative care and its impact on other forms and 3) develop recommendations to improve the acceptance and feasibility of level 2 palliative care. Therefore, the Polite study aims at investigating how (and to what extent) level 2 palliative care is integrated into end-of-life care in Germany, and delineating its general impact on patient care.

Specifically, the study aims at answering the following questions:

- Which level 2 palliative care components are applied in practice, and how?
- What are the experiences of care providers and policy makers with respect to level 2 palliative care? What do they identify as the barriers to and facilitators of level 2 palliative care, in practice?
- What adaptations to level 2 palliative care are reasonable and necessary to support its administration and optimise end-of-life care?

### Aims of the study protocol

The present protocol describes a mixed-methods study involving qualitative and quantitative methods and participatory aspects. The research aims at making an original contribution to the palliative care literature by increasing the transparency of the administration of outpatient palliative care in Germany. Publication of the study protocol represents the first step in this process. The authors believe that the project partners, scientists studying similar topics, and the broader scientific community will be interested in consulting the study protocol to obtain an overview of the project. Furthermore, health care insurance providers and policy makers will find the study design for systematic research on outpatient palliative care highly relevant.

## Materials and methods

Polite is an 18-month project funded by the innovation fund of the Federal Joint Committee (Grant N° 01VSF20028).

### Study design

**Fig 1:**
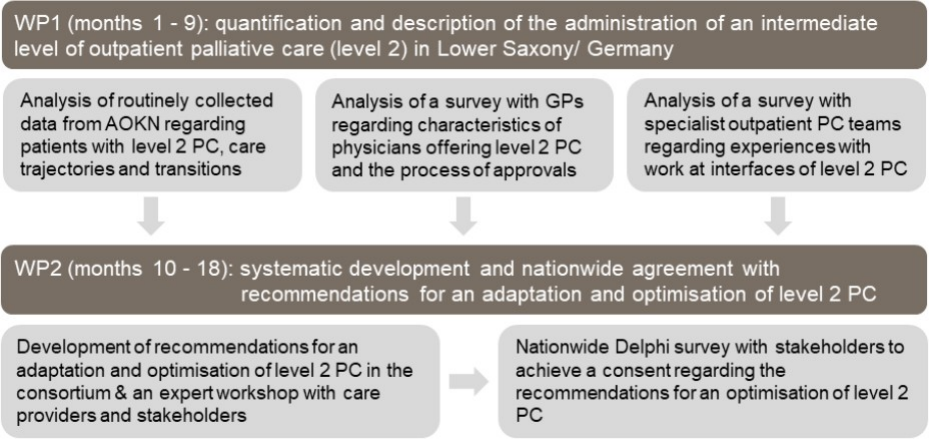
Mixed-methods study design. AOKN = statutory health insurance provider in Lower Saxony; GPs = general practitioners; PC = palliative care; WP = work package.

The multi-perspective, observational study will follow a mixed-methods approach, spread across two research phases (Fig 1).

### Setting

Study phase 1 will be conducted in Lower Saxony. Lower Saxony is the second largest federal state in Germany, comprised of both urban and rural areas. As its characteristics are similar to those of other federal states, the results of the research will be generalisable throughout Germany. Importantly, Lower Saxony includes urban areas with continuous GPs and palliative care providers, as well as rural areas with low population density and challenging health care structures.

### Study phases and data collection

In phase 1, work package 1a, quantitative routinely collected data from the statutory health insurance provider Allgemeine Ortskrankenkassen Lower Saxony (AOKN) will be used to analyse the development of level 2 outpatient palliative care services over time and to identify patients who died in 2019 and received outpatient palliative care services (at any level). Sociodemographic and disease-related data for these patients will be collected and compared according to those who received level 2 palliative care versus those who received palliative care at levels 1 or 3. The consortium will reveal the care trajectories of these patients, the patients who received different forms of outpatient palliative care and the transitions that occurred between levels of outpatient palliative care. In addition, the use of health care services beyond palliative care (but alongside level 2 palliative care) will be examined from billing records; such services might include medical specialists or hospital admissions. These data are expected to reveal the extent to which level 2 palliative care is implemented. Finally, a comparison with data from patients deceased in 2017 will show on a macro level whether (and to what extent) the introduction of level 2 palliative care shifted the type and administration of palliative care at levels 1 or 3. The data required for this will be extracted and analysed by the AOKN.

In phase 1, work package 1b, a quantitative, standardised questionnaire for GPs will be developed on the basis of the literature, previous scientific findings, available political statements and contributions from relevant professional associations. The online survey will be sent to all (N = 5,158) GPs registered in Lower Saxony to collect information on the number and characteristics of physicians offering level 2 palliative care. A response rate of approximately 15% (n = 774) is expected.

In phase 1, work package 1c, comparable to work package 1b, a quantitative, standardised online questionnaire for teams providing specialist outpatient palliative care (level 3) will be developed. The questionnaire will aim at quantifying respondents’ experiences with working at the interface of level 2 and level 3 palliative care. It will also seek to ascertain how work at the interfaces between palliative care levels 1, 2 and 3 does or does not succeed in practice, and which practical implementation models for level 2 have been developed. The questionnaire will be sent to all (N = 59) registered specialist outpatient palliative care teams in Lower Saxony, and a response rate of approximately 50% (n = 30) is expected.

In phase 2, work package 2a, the results from phases 1a–c will be presented and discussed in an expert workshop at Hannover Medical School. The discussion will aim at developing ideas to adapt and optimise level 2 outpatient palliative care. Approximately n = 20 care providers and stakeholders from hospice and palliative care in Germany will participate in the expert workshop. A drop-out rate of 10% due to illness, other commitments and personal reasons is expected. Personal invitations will be sent out until the target number of N = 22 experts is reached. The result of the work package 2a will be initial formulations of possible recommendations, which will be subjected to further scrutiny in work package 2b.

In phase 2, work package 2b, the empirically derived recommendations from phases 1 and 2a will be agreed upon via a multi-round Delphi survey [12] involving n = 40 experts with sufficient influence to promote the project results and recommendations nationally, including participants from phase 2a. To compensate for an expected participation rate of 20–30% and a drop-out rate of 10%, approximately N = 120 experts will be invited. Delphi survey data will be collected with the aid of an online tool. Participants will be asked to indicate their level of agreement with each individual recommendation on a 4-point Likert scale, with regards to relevance, clarity and feasibility. Free text fields will also be included for participants to provide additional comments on the recommendations.

The project team is presently developing the research infrastructure with respect to (e.g.) data protection, ethical approval, contracts with project partners and public relations to prepare for the project start. Moreover, strategies to support the development of address registers for GPs and specialist outpatient palliative care teams are being discussed.

### Study population

The main target groups for analysis will be:

1. AOKN members who died in 2019;
2. health care providers and experts at the micro level, including GPs; oncologists; pain medicine or palliative care specialists; geriatricians; and multi-disciplinary specialists from level 3 outpatient palliative care teams, nursing care services, nursing care facilities and inpatient hospices; and
3. palliative and hospice care stakeholders at the meso and macro levels, including representatives from the German Hospice and Palliative Care Association (Deutscher Hospiz- und Palliativverband), the German Association for Palliative Medicine (Deutsche Gesellschaft für Palliativmedizin), the Professional Association of specialised outpatient palliative care (Fachverband SAPV), other societies and professional associations, the Association of Statutory Health Insurance Physicians Lower Saxony (Kassenärztliche Vereinigung Niedersachsen), GP associations, the German Home Care and Nursing Society (Deutscher Pflegeverband e.V.), and health care insurance providers.

### Inclusion, exclusion and termination criteria

In phase 1, work package 1a, only secondary data will be analysed; thus, there will be no participant recruitment. Inclusion criteria will depend on the type of analysis (e.g. utilisation of level 2 outpatient palliative care or death). For deceased patients, the inclusion criteria will comprise: an age of 18 years or older at the time of death and continuous insurance during the year of death and the preceding calendar year.

In phase 1, work packages 1b and 1c, online surveys will be conducted with all registered GPs and all level 3 specialist outpatient palliative care teams in Lower Saxony. The aim will be to achieve a full assessment of both groups in this federal state. Therefore, no further inclusion or exclusion criteria will be needed. Each survey participant will independently decide whether to participate, as well as when to cease participation, if desired. No termination criteria will be defined.

In phase 2, work packages 2a and 2b, experts will participate in a workshop and/or Delphi survey. Participants will be selected according to the principle of diversity, in order to ensure the adequate representation of inpatient, ambulatory, generalist and specialist service providers and all interfaces with level 2 palliative care. The exclusion criteria will be comprised of insufficient German language skill and less than 1 year of experience in hospice and palliative care. Experts will be able to terminate their participation at any time.

### Data analysis

The data analysis will aim at describing the current state of level 2 outpatient palliative care administration, in practice, and determining consented recommendations for its optimisation and development. The analysis of secondary data from the AOKN in phase 1, work package 1a, will be carried out quantitatively and descriptively. Quantitative data from the surveys with GPs and specialist outpatient palliative care teams in work packages 1b and 1c will also be analysed descriptively, using IBM SPSS Statistics version 25 (SPSS Inc., Chicago, IL, USA).

Qualitative data from the expert workshop in phase 2, work package 2a, will be transcribed verbatim from the audio record by student assistants, and analysed qualitatively in MaxQDA (VERBI Software Consult Sozialforschung GmbH, 1989–2020), using the methodological principles of qualitative content analysis [13, 14]. The thematic characteristics of level 2 palliative care determined in previous study phases will comprise an a priori category system, which will be successively expanded with the workshop data.

Quantitative data from phase 2, work package 2b, will be processed in IBM SPSS Statistics 26 (SPSS Inc., Chicago, IL, USA). Recommendations from the first Delphi round that receive support from at least 80% of the participants (on the basis of the scale responses “I rather agree” and “I fully agree”) will be considered consented. Results will be calculated by means of a frequency analysis. Recommendations that are non-consented in Delphi round 1 will be revised according to any free text comments and subjected to a second Delphi round. This interim analysis and adaptation of the recommendations will take approximately 2–3 weeks. Following this, the revised recommendations will be sent to all participants who completed the first Delphi round for a second online evaluation. A third round will be applied, if necessary, following the same method.

### Data security

All personal data will be treated in accordance with the German General Data Protection Regulation. All data collected in the course of the study will be subject to confidentiality and all members of the project team will be bound to data secrecy. The data collected from all work packages will be anonymised and stored in accordance with the applicable data protection guidelines. Furthermore, they will be exclusively analysed with regard to the objectives stated in the project proposal. The quantitative survey data will be anonymised for the statistical analysis. The analysis of all qualitative data will be carried out in pseudonymised form (i.e. without the names of persons, institutions or locations). Recorded data will be deleted upon participants’ request, as long as the data have not yet been anonymised.

### Expected results

The project team assumes that: 1) health care providers and stakeholders will report different experiences, both positive and negative, with the provision of level 2 palliative care; 2) experience-based ideas from health care providers and stakeholders could contribute to optimising level 2 palliative care; and (3) the care of seriously ill patients in an outpatient setting could be improved by the empirical results of a systematic evaluation of the administration of level 2 palliative care.

The main expected results are as follows: 1) a multi-perspective overview of the extent and nature of the administration of level 2 outpatient palliative care in Lower Saxony and 2) a set of evidence-based recommendations for the needs-oriented development of level 2 palliative care in Germany.

### Ethical considerations

The consortium management has submitted the study protocol to the responsible ethics committee and made revisions in accordance with their advice. A positive ethics vote (N° 9629_BO_K_2021) for Polite was issued by the Ethics Committee of the Hannover Medical School on 23 February 2021. The data protection officer of the MHH and the AOKN confirmed that the study meets all data protection laws.

All participating health care providers and stakeholders from hospice and palliative care will be informed (verbally and in writing) in detail about the questionnaires and the aims and purpose of the project, in advance. Participation will only commence following participants’ express consent to participate in the online survey (phase 1, work packages 1b and 1c; phase 2, work package 2b) or give additional written informed consent (phase 1, work packages 1b and 1c; phase 2, work package 2a). Written information will be provided to all participants. Each participant will have the right to refuse or terminate participation at any time before or during the data collection, without giving a reason.

## Discussion

### Study risks

Access to health care providers and stakeholders in the field of hospice and palliative care can be difficult, depending on the attitudes and experiences of these parties with respect to scientific research. Thus, recruiting the intended number of participants in phase 1, work packages 1b and 1c, and phase 2, work packages 2a and 2b, may be methodologically difficult. However, there are several reasons to predict the successful application of the various work packages: 1) participation will require only a single (questionnaire, expert workshop) or a few short (Delphi survey) expenditures of time; 2) the target populations are not difficult to reach; 3) the target number of participants was calculated in accordance with the topics and research methods applied in the respective study phases; and 4) recruitment will take place throughout Lower Saxony in phase 1 and Germany in phase 2.

To achieve the target sample sizes, the project team will take the following actions, if necessary:

Phase 1:
  - All registered GPs in phase 1, work package 1b, will be informed about the project and invited to participate in the survey, primarily via email and secondarily via letter or fax, if no email address is available.
  - The survey invitation will be advertised in the ‘Ärzteblatt’ Lower Saxony, which is distributed to all registered physicians in Lower Saxony. GPs on existing mailing lists (e.g. for teaching practices at the Hannover Medical School) will also be contacted.
  - All specialised palliative care teams in phase 1, work package 1c, will be informed about the project and invited to participate in the online survey via email or, in exceptional cases, via letter.
  - The online questionnaires for work packages 1b and 1c will be accessible for at least 4 weeks, with weekly reminders sent via email.
Phase 2:
  - To encourage participation in the planned expert workshop in phase 2, work package 2a, participants will be compensated for their loss of working hours, travel costs to and from the workshop and overnight accommodation.
  - To compensate for a possible low participation rate of 20–30% and an expected drop-out rate of 10% during the Delphi survey in phase 2, work package 2b, as many potential participants as possible will be informed and invited to participate.
  - It is assumed that, based on their experiences with palliative care and health services research, participating health care providers and stakeholders will have a high intrinsic motivation to improve palliative care.
  - The project team will respond to challenges in a timely manner, organising additional conference calls and project meetings, as needed.

### Dissemination and implementation

The results of the project will facilitate the nationwide optimisation of outpatient palliative care, as well as its administration. The findings may also be used to improve hospice and palliative care and propose structural changes to the legal framework. The empirically developed recommendations for optimising level 2 palliative care may be used to inform the legislative adaptation of level 2 palliative care. This would enable a rapid transfer into practice for the benefit of patient care, impacting a very large population. Good outpatient palliative care–and especially early palliative care–can allow patients to die at home and avoid hospitalisation and cost-intensive therapies; at the same time, it can lead to a higher quality of life and better symptom control at the end of life [15].

All project partners support the dissemination of the recommendations following project completion. To promote the accessibility and long-term safeguarding of the research data and results for the scientific community, the consortium will report comprehensively and transparently on the project and, regardless of the results, prepare national and international publications (with open access, where possible). Furthermore, the project team will make electronic research data (protected by data protection and copyright) available for secondary use in response to verifiably justified requests.

## Conclusion

The present study protocol explains the purpose, significance and scope of the mixed-methods study Polite, as well as the study design. The empirically developed recommendations that will be generated in this study are expected to optimise outpatient palliative care in Germany. The results may also initiate structural changes to the legal framework.

The authors’ goal of publishing the present study protocol is to promote research transparency beyond registration in the German Clinical Trials Register. Moreover, the study protocol may act as a point of reference for the Polite project team, the scientific community and other parties interested in the scientific and ethical aspects of the study. Finally, the publication of the study protocol may prevent unnecessary duplication.
